# Use of Partial Splenic Embolization Following a Transjugular Intrahepatic Portosystemic Shunt to Reduce Persistent Portal Hypertension Sequelae

**DOI:** 10.7759/cureus.89540

**Published:** 2025-08-07

**Authors:** Gunnar Schultz, Mohamed Naas, Jay Webb

**Affiliations:** 1 Radiology, Florida State University College of Medicine, Pensacola, USA; 2 Radiology, Florida State University College of Medicine, Tallahassee, USA; 3 Interventional Radiology, Sacred Heart Hospital, Pensacola, USA

**Keywords:** ir-guided embolization, liver cirrhosis, portal hypertension, splenomegaly, transjugular intrahepatic portosystemic shunt (tips)

## Abstract

Transjugular intrahepatic portosystemic shunt (TIPS) placement is a well-established intervention for portal hypertension. However, some patients experience persistent complications such as encephalopathy, ascites, or thrombocytopenia, especially when further TIPS optimization is not technically possible. Partial splenic embolization (PSE), typically performed for hypersplenism or certain hematologic conditions, can reduce portal venous inflow and improve cytopenias; however, its use as an adjunct to TIPS is less well described. We present the case of a 73-year-old male with cirrhosis secondary to hepatitis C who developed recurrent ascites, worsening encephalopathy, and refractory thrombocytopenia following portal vein recanalization with TIPS placement 18 months prior. The patient’s TIPS could not be further optimized due to technical limitations. Imaging demonstrated marked splenomegaly and an enlarged splenic artery, suggesting increased splenic contribution to portal inflow. PSE of the lower pole was performed. Post-procedure, the patient experienced significant clinical improvement. Encephalopathy episodes resolved, no ascites was seen on follow-up CT, and platelet count rose from <50,000/µL to >100,000//µL. The patient subsequently developed a splenic abscess requiring percutaneous intervention. PSE may serve as an effective adjuvant to TIPS in selected patients with persistent portal hypertension and cytopenias when further TIPS optimization is not possible. This case highlights the potential for PSE to reduce portal inflow and improve platelet count, suggesting an expanded role in the management of complex portal hypertension.

## Introduction

Liver cirrhosis is characterized by diffuse fibrosis and disorganized regeneration of hepatocytes, leading to nodule formation and increased intrahepatic vascular resistance [1,2]. This process elevates portal venous pressure, resulting in complications such as ascites, variceal hemorrhage, hepatic encephalopathy, and thrombocytopenia due to hypersplenism [2].

The transjugular intrahepatic portosystemic shunt (TIPS) procedure is widely used to lower portal hypertension and its sequelae [3,4]. At our institution, all TIPS procedures utilize Viatorr stent-grafts, which can be maximally ballooned to 10 mm in diameter. Once maximally dilated, further optimization of the shunt is generally not feasible except by placing a parallel TIPS, extending the stent within the portal system, or, rarely, considering a direct intrahepatic portosystemic shunt. In some cases, however, anatomic or technical limitations preclude additional shunt intervention.

Partial splenic embolization (PSE) is most commonly used to treat hypersplenism, particularly in patients with cirrhosis and secondary cytopenias or in certain hematological conditions [5,6]. By reducing the volume of functional splenic tissue, PSE decreases splenic sequestration of platelets and other blood cells and potentially reduces portal venous inflow [5,6]. Although there is robust literature describing PSE for cytopenias and post-liver transplant patients with persistent portal hypertension, its role as an adjuvant to TIPS in non-transplant cirrhotic patients remains less well described [7,8].

Here, we present the case of a patient with cirrhosis who underwent portal vein recanalization and TIPS for recurrent variceal bleeding and ascites. Despite a technically successful intervention, he developed persistent complications, including recurrent ascites, worsening encephalopathy, and thrombocytopenia, with no options for further TIPS optimization. This case highlights the utility of PSE as a means to decrease portal inflow and improve clinical outcomes in complex, refractory portal hypertension.

## Case presentation

A 73-year-old male with cirrhosis secondary to cured hepatitis C presented with two days of worsening confusion and lethargy. Eighteen months earlier, he had undergone portal vein recanalization with TIPS placement for recurrent variceal hemorrhage and ascites. Since then, he began to experience occasional episodes of hepatic encephalopathy. Ascites accumulation was markedly decreased, but he still required occasional paracentesis. He also had chronic thrombocytopenia with a platelet count typically around 30,000/µL. This was presumably a contributing factor to a chronic subdural hematoma, which had been treated with middle meningeal artery embolization in the interim.

On presentation, the patient was lethargic but arousable, oriented only to person, without focal neurologic deficits. Laboratory evaluation showed markedly elevated ammonia (230 µmol/L; reference: 18-72µmol/L), thrombocytopenia (platelet count: 37 × 10^3^/µL), anemia (hemoglobin: 8.6 g/dL), and mild coagulopathy (prothrombin time: 19.9 seconds, international normalized ratio: 1.7), consistent with his known baseline. Renal function revealed an acute kidney injury on chronic kidney disease stage 3, which resolved with intravenous fluids.

Given his history and presentation, hepatic encephalopathy was suspected and managed with rectal lactulose and rifaximin, resulting in a decrease in ammonia levels to 74-106 µmol/L. We were consulted to evaluate for potential optimization of his TIPS. However, further TIPS optimization or placement of a second TIPS was not feasible due to the prior portal vein recanalization, maximal stent dilation, and lack of further access.

**Figure 1:**
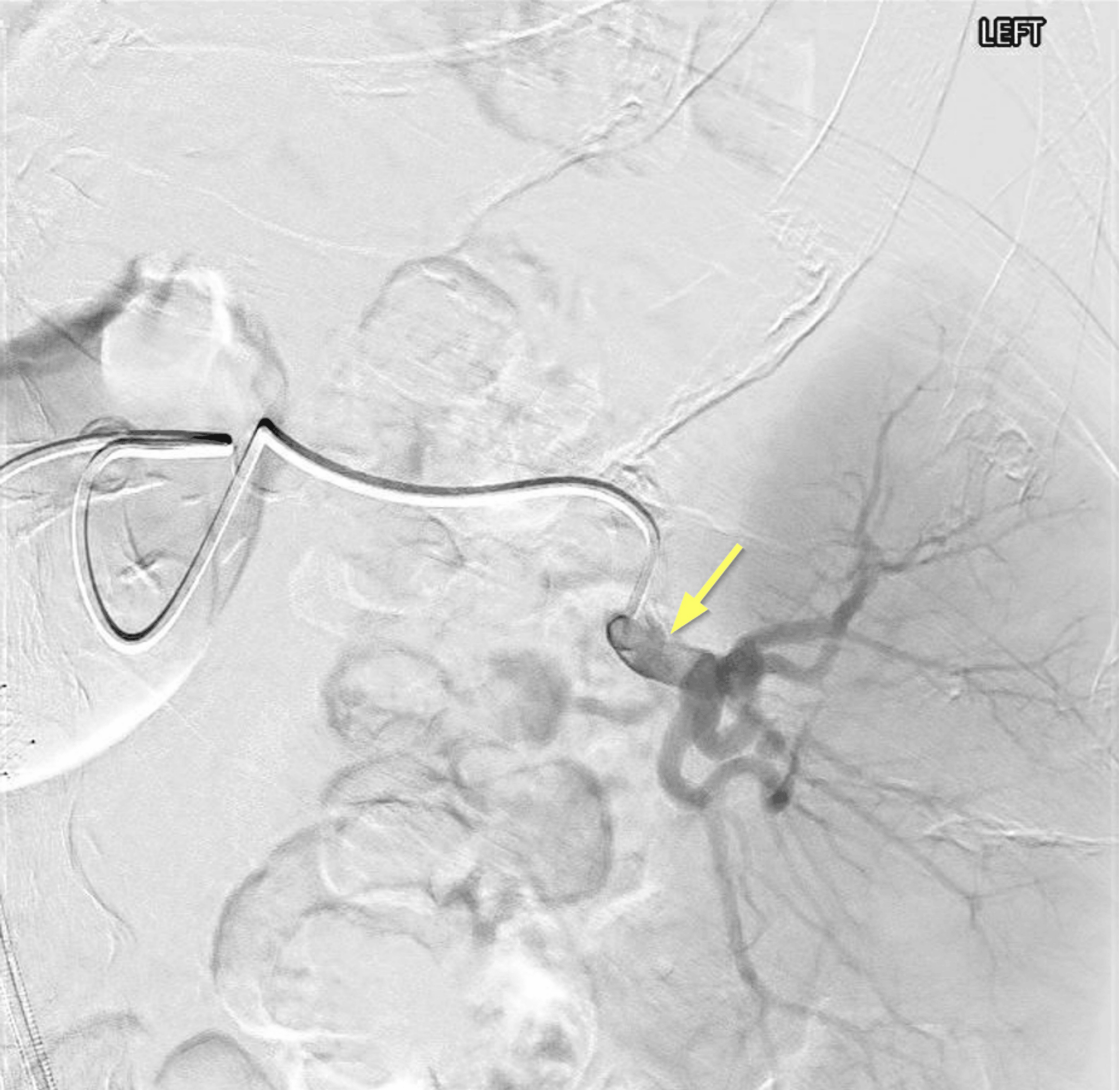
Angiography of the splenic artery before partial splenic embolization. Pre-embolization angiogram shows perfusion of the entire spleen, with the upper pole of the splenic artery supplying more than 50% of the parenchyma and the lower pole supplying just under half (yellow arrow).

**Figure 2:**
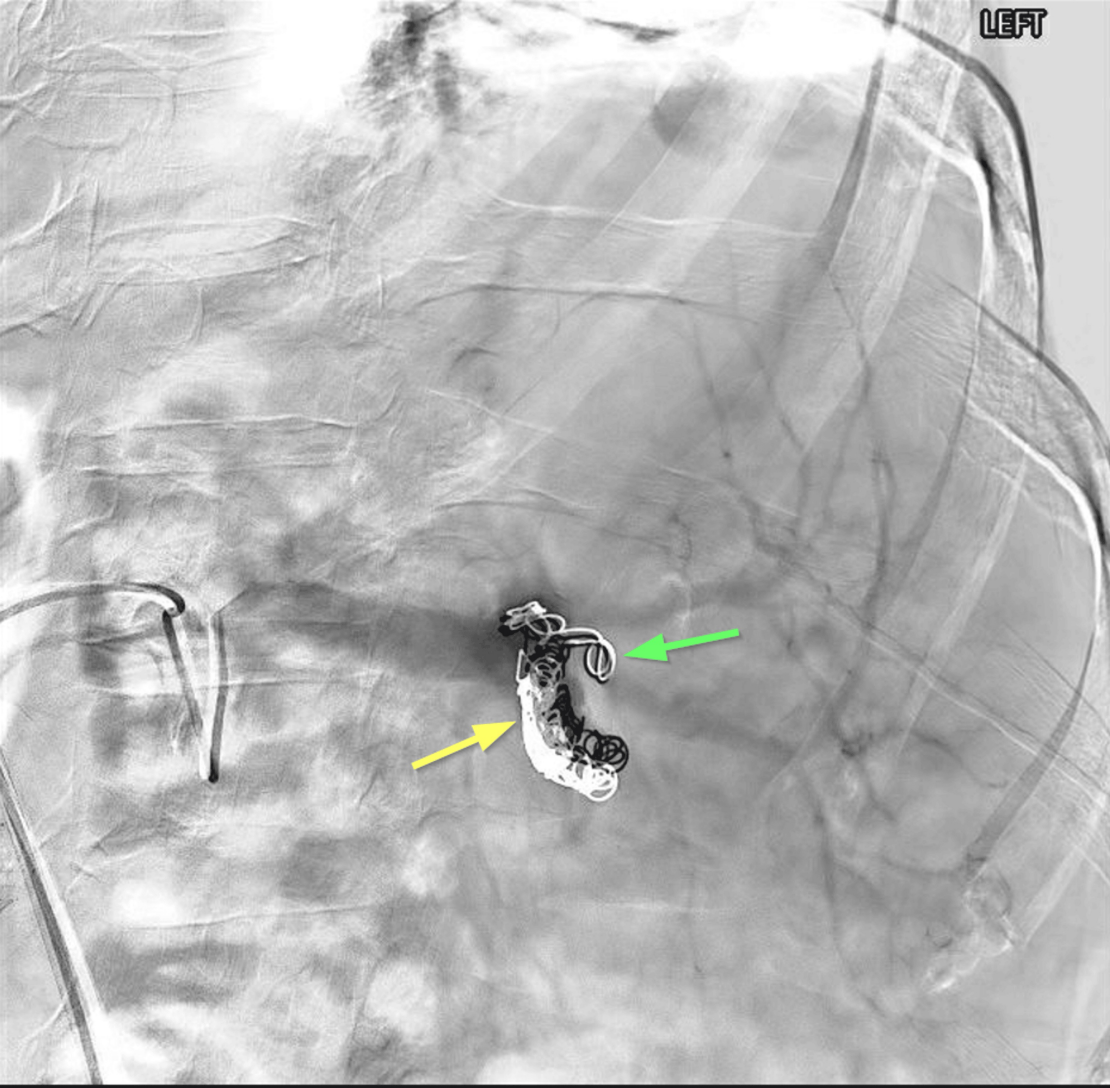
Angiography of the splenic artery after partial splenic embolization. Post-embolization angiogram demonstrates successful occlusion of the lower branch of the splenic artery with minimal perfusion (yellow arrow). The upper branch displays preserved perfusion. The tail of the final coil extends into one of the upper pole branches, but does not appear significantly occluded (green arrow).

Abdominal imaging demonstrated marked splenomegaly and an enlarged splenic artery, suggesting a significant splenic contribution to portal venous inflow. The decision was made to proceed with PSE to reduce portal inflow and potentially improve platelet count. PSE was performed via femoral arterial access. Our method for estimating the volume of spleen to be embolized was entirely visual, based on frontal angiography; more accurate techniques (such as cone-beam CT with selective angiography) were not possible due to the patient’s body habitus and equipment limitations. Angiography revealed that the upper pole of the splenic artery supplied more than 50% of the parenchyma, and the lower pole just under half (Figure 1). The lower pole was targeted for embolization, using one vial each of 300-500 µm and 500-700 µm Embospheres, followed by several 8 mm and 6 mm Nester coils to prevent reperfusion (Figure 2).

During coil deployment, the tail of the final coil inadvertently extended into a branch of the upper pole splenic artery, though this did not appear completely occlusive. However, follow-up CT revealed that this branch was ultimately occluded, resulting in greater than planned infarction.

**Figure 3:**
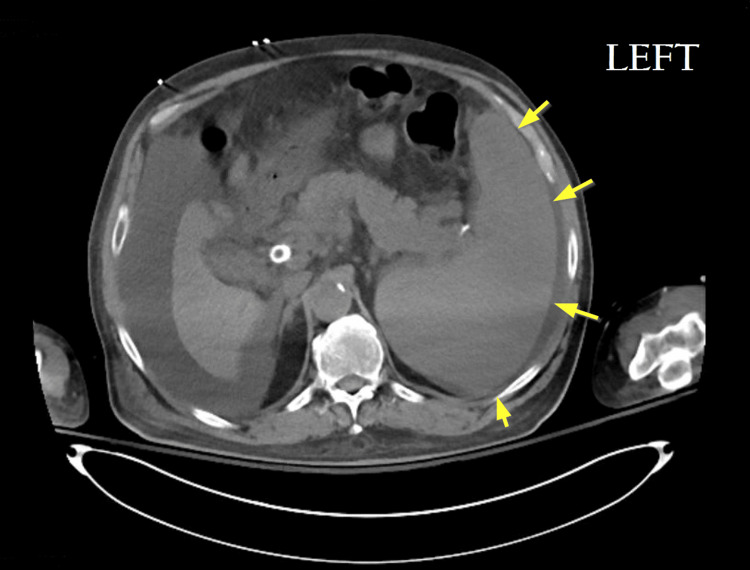
Axial CT image of the spleen before partial splenic embolization. Pre-embolization CT demonstrates splenomegaly with estimated splenic dimensions of 14 × 19 × 19 cm and a calculated total volume of approximately 3,032 mL (yellow arrows).

**Figure 4:**
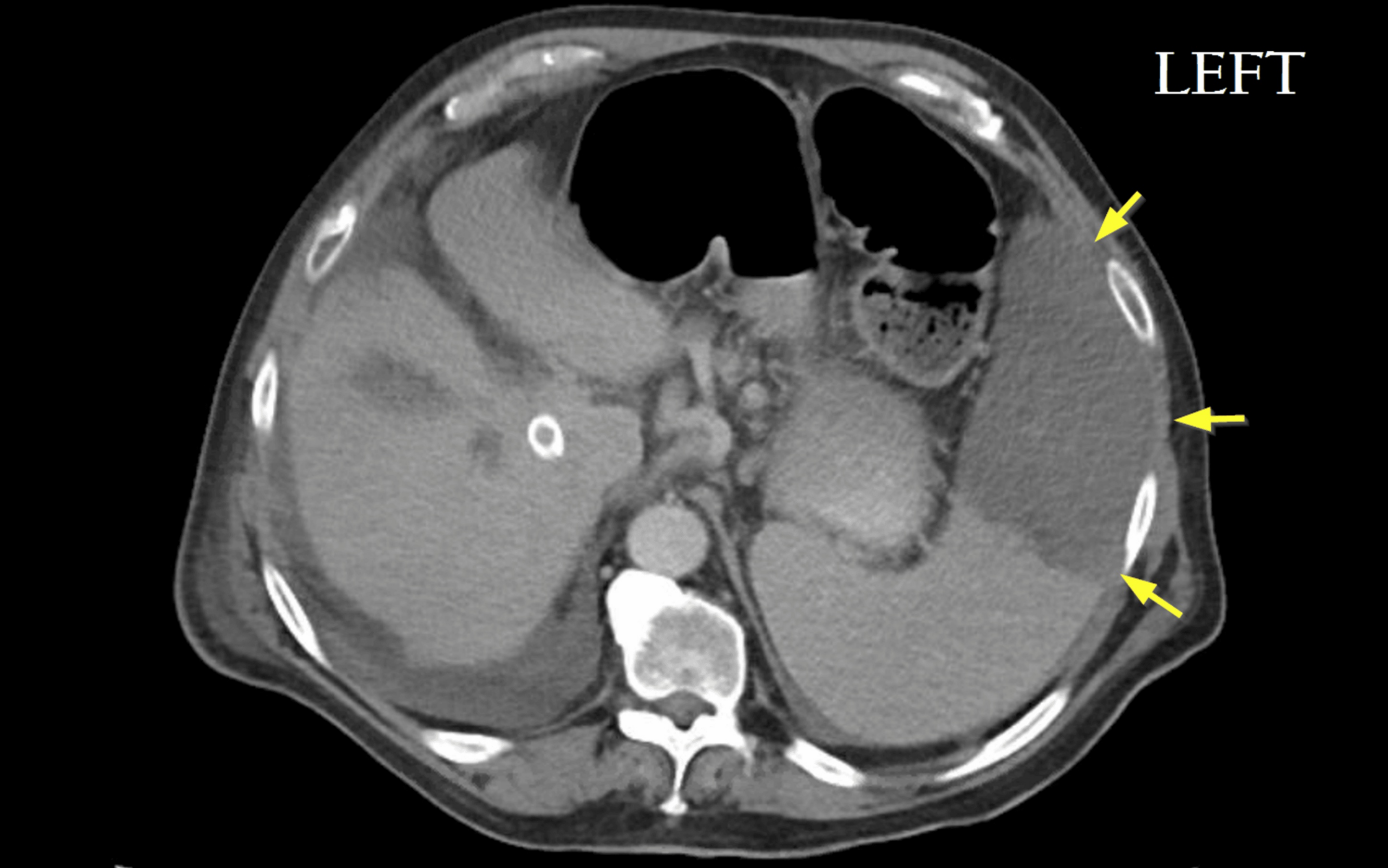
Axial CT image of the spleen after partial splenic embolization. Post-embolization CT demonstrates a reduced splenic size with an area of infarct inferiorly and anteriorly (yellow arrows). Approximate active tissue dimensions post-embolization were 9 × 12 × 14 cm, with a calculated total volume of 907 mL.

Splenic size and volume were estimated using cross-sectional measurements and a correction factor of 0.6 to account for splenic shape. Pre-embolization CT showed splenomegaly with dimensions 14 × 19 × 19 cm and a calculated volume of approximately 3,032 mL (Figure 3). Post-embolization, active splenic tissue measured 9 × 12 × 14 cm (volume: 907 mL), a 70% reduction (Figure 4), exceeding the targeted 50% due to the additional infarction described.

The patient’s mental status improved over several days, and he was discharged. Laboratory values from presentation, pre-PSE, and one month post-PSE are shown in Table 1.

**Table 1:** Laboratory values on presentation, pre-PSE, and post-PSE. AST = aspartate aminotransferase; ALT = alanine aminotransferase; ALP = alkaline phosphatase; PT = prothrombin time; INR = international normalized ratio; BUN = blood urea nitrogen; PSE = partial splenic embolization

Parameter (unit)	Reference range	On presentation	Pre-PSE	Post-PSE
ALT (U/L)	7–56	20	16	14
AST (U/L)	10–40	53	33	35
ALP (U/L)	44–147	89	69	102
Total bilirubin (mg/dL)	0.1–1.2	1.3	1.7	1.4
PT (seconds)	11–13.5	19.9	24.1	18.0
INR	0.8–1.2	1.7	2.2	1.5
Ammonia (µmol/L)	18–72	230	79	84
Platelets (×10³/µL)	150–400	37	40	124
Hemoglobin (g/dL)	13.8–17.2	8.6	8.7	10.8
Hematocrit (%)	40.7–50.3	25.7	27.2	32.2
BUN (mg/dL)	7–20	23	13	25
Creatinine (mg/dL)	0.6–1.3	1.97	1.46	1.98

Approximately one month after the procedure, the patient was readmitted with fever, leukocytosis, and left upper quadrant pain. CT revealed that the infarcted portion of the spleen had liquefied, enlarged, and developed capsular thickening. A percutaneous drain was placed, and fluid cultures grew *Staphylococcus epidermidis*. The drain remained in place for about a month, then was removed after resolution of the collection. The patient recovered without further complications.

On follow-up, the patient’s encephalopathy episodes were markedly decreased, and both clinical examination and serial CT imaging demonstrated no ascites. Platelet count increased from typically around 30,000/µL pre-PSE to consistently over 100,000/µL post-PSE.

## Discussion

While TIPS is effective in lowering portal venous pressure and reducing complications of portal hypertension, some patients experience persistent portal hypertension and its sequelae, as well as an increased incidence of hepatic encephalopathy [4,9]. In our patient, recurrent ascites, encephalopathy, and thrombocytopenia persisted despite technically successful TIPS, and further shunt optimization was not possible due to prior portal vein recanalization and stent limitations.

Hepatic encephalopathy after TIPS arises from increased shunting of portal blood, rich in ammonia and other neurotoxins, into the systemic circulation, bypassing hepatic metabolism [9-11]. The rationale for partial splenic embolization in this context was twofold: (1) to decrease portal inflow from the spleen, potentially improving the effectiveness of hepatic filtration for blood draining the intestines, and (2) to improve hypersplenism-related cytopenias, particularly thrombocytopenia [5,6,12].

PSE is an established therapy for hypersplenism, improving cytopenias in cirrhotic and select hematologic patients [5,6,12]. However, its use for reducing portal venous inflow and thereby addressing refractory portal hypertension in non-transplant patients is less frequently described in the literature [7,8]. In this case, PSE was associated with clear clinical benefit: the patient’s episodes of hepatic encephalopathy decreased, ascites was absent both clinically and on serial CT imaging, and platelet counts increased substantially.

A notable technical aspect of this case was the estimation of the splenic volume to be embolized. Our approach was visual, based on frontal angiography, due to the patient’s body habitus and cone-beam CT equipment limitations. More precise assessment with cross-sectional or cone-beam imaging may allow for improved volume targeting in future cases.

Optimal infarct volume for PSE remains debated. The general consensus is that infarcting 30-50% of splenic parenchyma achieves benefit while minimizing risk [13,14]. In our case, inadvertent extension of a coil into the upper pole led to approximately 70% infarction, which likely contributed to the development of a splenic abscess, a well-recognized complication of more extensive splenic infarction [13]. Notably, this could likely have been prevented by using detachable coils, but would have increased cost and procedure time significantly. Management included percutaneous drainage and antibiotics, resulting in full recovery.

Our experience suggests that, particularly in patients with large spleens, a staged approach to PSE may be prudent to limit complications. Most patients after PSE experience post-embolization syndrome, characterized by fever, nausea, and left upper quadrant pain [14]. The majority of less common complications, including abscess, pleural effusion, pneumonia, pulmonary embolism, and portal vein thrombosis, occurred when embolization volume was 70% or higher [14]. Ultimately, this case supports consideration of PSE as a useful adjunct in patients with persistent portal hypertension and cytopenias when further TIPS optimization is not possible, provided that procedural risks are carefully weighed and mitigated.

## Conclusions

This case highlights the value of PSE as an adjunctive therapy in the management of persistent portal hypertension when further TIPS optimization is not feasible. In our patient, PSE was performed primarily to decrease portal venous inflow and improve refractory symptoms, resulting in the resolution of ascites, improved encephalopathy, as well as a marked improvement in platelet count. Although complications such as splenic abscess may occur, particularly with more extensive infarction, PSE can offer significant clinical benefit in carefully selected patients with complex portal hypertension.
